# S100B Protein, Brain-Derived Neurotrophic Factor, and Glial Cell Line-Derived Neurotrophic Factor in Human Milk

**DOI:** 10.1371/journal.pone.0021663

**Published:** 2011-06-27

**Authors:** Ruisong Li, Wei Xia, Zhihong Zhang, Kun Wu

**Affiliations:** 1 Department of Nutrition and Food Hygiene, School of Public Health, Harbin Medical University, Harbin, China; 2 Department of Children Health and Hygiene, School of Public Health, Harbin Medical University, Harbin, China; VIB&Katholieke Universieit Leuven, Belgium

## Abstract

**Background:**

Human milk contains a wide variety of nutrients that contribute to the fulfillment of its functions, which include the regulation of newborn development. However, few studies have investigated the concentrations of S100B protein, brain-derived neurotrophic factor (BDNF), and glial cell line-derived neurotrophic factor (GDNF) in human milk. The associations of the concentrations of S100B protein, BDNF, and GDNF with maternal factors are not well explored.

**Methodology/Principal Findings:**

To investigate the concentrations of S100B protein, BDNF, and GDNF in human milk and characterize the maternal factors associated with their levels in human milk, human milk samples were collected at days 3, 10, 30, and 90 after parturition. Levels of S100B protein, BDNF, and GDNF, and their mRNAs in the samples were detected. Then, these concentrations were compared with lactation and other maternal factors. S100B protein levels in human milk samples collected at 3, 10, 30, and 90 d after parturition were 1249.79±398.10, 1345.05±539.16, 1481.83±573.30, and 1414.39±621.31 ng/L, respectively. On the other hand, the BDNF concentrations in human milk samples were 10.99±4.55, 13.01±5.88, 13.35±6.43, and 2.83±5.47 µg/L, while those of GDNF were 10.90±1.65, 11.38±1., 11.29±3.10, and 11.40±2.21 g/L for the same time periods. Maternal post-pregnancy body mass index was positively associated with S100B levels in human milk (r = 0.335, P = 0.030<0.05). In addition, there was a significant correlation between the levels of S100B protein and BDNF (z = 2.09, P = 0.037<0.05). Delivery modes were negatively associated with the concentration of GDNF in human milk.

**Conclusions:**

S100B protein, BDNF, and GDNF are present in all samples of human milk, and they may be responsible for the long term effects of breast feeding.

## Introduction

The S100B protein is a member of the calcium-binding S100 family, which is characterized by a low molecular weight and a special EF-hand structure [1]. Like most members of this family, S100B has a homodimeric structure wherein each beta monomer is approximately 10.5 kDa. Each monomer has two EF hand sites for Ca2+ binding and independent sites for Zn2+ binding. It has two disulfide bridges, but the dimeric structure is maintained independently of this aspect.

Brain-derived neurotrophic factor (BDNF) is a small dimeric protein belonging to the neurotrophin family, which is widely expressed in the mammalian adult brain [2].

Glial cell line-derived neurotrophic factor (GDNF) is a distant member of the transforming growth factor β superfamily that was originally isolated from the rat B49 glial cell line [3]. This protein is a glycosylated, disulfide-bonded homodimerwith a molecular weight of 33–45 kDa. Its monomer has a molecular weight of 16 kDa after deglycosylation [4].

S100B, BDNF, and GDNF play a critical role in the development and maintenance of the nervous system, and in neuronal survival and proliferation [3], [5]–[19]. These proteins have been implicated in the modulation of learning and memory [20]–[24]. Human milk protects the infants from infection, modulates their immune function, and affects their overall development [25]. The present study investigates the concentration of S100B, BDNF, and GDNF in the milk of Chinese women after parturition to clarify the function of these cytokines.

## Methods

### Ethics Statement

Approval from the Ethical Committee of the Harbin Medical University was obtained prior to this study. Written informed consent was obtained before collection of milk samples from donors.

### Participants

Samples for ELISA analysis were collected from 42 mothers: 31 of whom had abdominal delivery at term, while 11 delivered vaginally at term. Milk samples were collected at 3, 10, 30, and 90 d after parturition. Participants who had gestational hypertension, diabetes, infection, fever, metabolic diseases, breast diseases, central nervous system diseases, malnutrition, maternal allergy, fetal anomaly, and smoking habits were excluded.

Human milk was collected by hand into sterile 5-ml Eppendorf tubes. Upon collection, samples were refrigerated at 4°C in a polystyrene box containing ice. All samples were immediately transferred to the laboratory, where they were stored at −80°C.

### ELISA analysis

After thawing at room temperature, milk samples were centrifuged at 1000 g for 10 min at 4°C. The supernatant was removed and re-centrifuged at 10,000 g for 30 min at 4°C, and the floating lipid layer and cellular sediments were removed. BDNF and GDNF concentration were measured in all samples by enzyme-linked immunosorbent assay (ELISA; R&D Systems, Inc., United States of America) according to the manufacturer's instructions. ELISA (Wuhan EIAab Science Co., Ltd., China) was also used to determine the concentration of S100B protein. All samples were tested in duplicate and the averages were reported. Intra-assay and the inter-assay variation coefficients were <5% and <10%, respectively. The assay ranges of the S100B protein, BDNF and GDNF ELISA kits were 15.6–1000 ng/L, 1.5–110 g/L, and 2–60 g/L, respectively.

### Western blot analysis

Protein concentrations were determined using the Lowry method of protein assay [26] with bovine serum albumin as standard. About10 µL of human milk (1000 g supernatant) were separated on 15% SDS–PAGE and transferred to a nitrocellulose membrane. Immunoblotting was performed using rabbit BDNF and GDNF antibodies (Wuhan Boster Bioligical Technology.,LTD, China). The membrane was then incubated with the secondary alkaline phosphatase-conjugated IgG and detected with the Western Blue Stabilized Substrate for alkaline phosphatase (Promega).

### RT-PCR analysis

Milk samples for reverse transcription-PCR (RT-PCR) analysis were collected from a mother at 3, 10, 30, and 90 d after parturition. The milks (15 ml) were centrifuged at 1000 g for 10 min at 4°C, and the RNA was extracted from the cell-pellet using TRIzol reagent (Invitrogen, Carlsbad, CA). RT-PCR (RNA PCR kit, TaKaRa Shuzo Co., Ltd., Japan) was conducted according to the manufacturer's manual. The quality of RNA extract was determined using the A260/A280 ratio, and was found to be 1.7–2.0 for all RNA preparations. A 1 μg portion of the total RNA was used for cDNA synthesis by reverse transcription (RT) with a final reaction mixture volume of 20 μl. RT was performed using a thermal program of 25°C for 10 min, 42°C for 30 min, and 95°C for 5 min. The cDNA was stored at −80°C for further use.

A 1.6 μl aliquot of the cDNA solution was used for the PCR assay (20 μl final volume). Samples were subjected to 36 cycles of PCR amplification: each cycle consisting of denaturation at 94°C for 30 s, annealing at a specified temperatures for 30 s, and extension at 72°C for 30 s. A final extension was performed at 72°C for 10 min.

Annealing temperatures (AT) and primer sequences are as follows: β2-actin (forward: 5′-CTCGCTGTCCACCTTCCA-3′; reverse: 5′-GCTGTCACCTTCACCGTTC-3′; size: 256 bp; AT: 56°C), S100B [27] (forward: 5′- CATTTCTTAGAGGAAATC-3′; reverse: 5′-ATGTTCAAAGAACTCGTG-3′; size: 147 bp; AT: 46°C), BDNF (forward: 5′-CAAACATCCGAGGACAAG-3′; reverse: 5′- GCCGTTACCCACTCACT-3′; size: 379 bp; AT: 56°C), and GDNF (forward: 5′- ACTTGGGTCTGGGCTATGAA-3′; reverse: 5′-TGTCACTCACCAGCCTTCTATT-3′; size: 132 bp; AT: 53°C). Amplification products were examined by electrophoresis on 1.5% agarose gel stained with ethidium bromide. All assays were performed with at least one replicate. The amplicons were matched with DL500 DNA Marker 100T (TaKaRa Shuzo Co., Ltd., Japan).

### Statistical analysis

All data were expressed as the mean ±SD and were analyzed using Stata version 10.0 (StataCorp, United States of America). Statistical analysis was performed using the generalized estimating equation. A linear correlation was conducted to assess the relationship of S100B milk concentrations and the body mass index (BMI) of mothers. Statistical significance was indicated by P values less than 0.05.

## Results

Mothers who participated in the study ranged from 19 to 38 years (mean age 25.26 years), with BMIs ranging from 21.7 to 34.8 (mean 27.57 kg/m^2^) and Gestational Ages between 37 and 42 weeks (mean 38.98 weeks). All mothers had their first accouchements at the time of the study and all showed normal clinical conditions.

Cytokine concentrations in the milk of the mothers, who gave samples at 30 (n = 40) and 90 (n = 24) d after parturition, are presented in Tables 1 and 2.

**Table 1:** Cytokines in the human milk from Chinese women during day 3, 10, and 30 after parturition (n = 42).

Cytokines	day 3	day 10	day 30*	*P*
S100B (ng/L)	1249.79±398.10	1345.05±539.16	1481.83±573.30	0.034
BDNF (µg/L)	10.99±4.55	13.01±5.88	13.35±6.43	0.205
GDNF (µg/L)	10.90±1.65	11.38±1.78	11.29±3.10	0.831

*number of donors equal to 40.

**Table 2:** Cytokines in human milk from Chinese women during day 3, 10, 30, and 90 after parturition (n = 24).

Cytokines	day 3	day 10	day 30	day 90	*P*
S100B (ng/L)	1221.63±338.27	1246.08±542.27	1381.28±525.48	1414.39±621.31	0.434
BDNF (µg/L)	10.64±5.402811	11.69±5.51	12.42±6.27	12.83±5.47	0.293
GDNF (µg/L)	10.95±1.87	11.37±1.93	11.47±2.65	11.40±2.21	0.735

**Figure 1:**
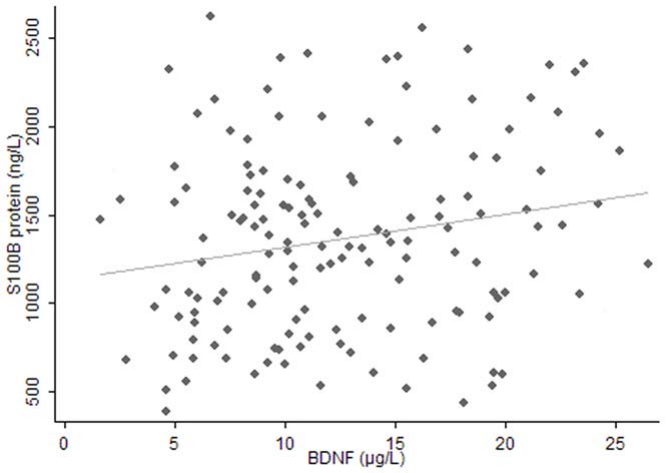
Positive association between levels of S100B and BDNF in human milk. The solid line represents the predicted regression line determined from repeated-measures analysis of S100B and BDNF concentrations 3 month after parturition. n = 24, P = 0.037<0.05.

S100B protein, BDNF, and GDNF were present in all samples of human milk. The levels of S100B protein peaked at 30 d after parturition , while BDNF and GDNF levels did not show variations with time. A significant correlation was found between S100B protein and BDNF levels at the third month after birth (z = 2.09, P = 0.037<0.05,Figure 1).

**Figure 2:**
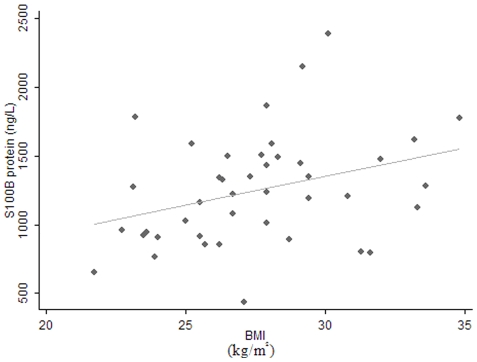
S100B levels in human milk were closely correlated with BMI. The solid line represents the predicted regression line determined from measures analysis of human milk collected 3 days after birth. Pearson correlation coefficient (*r*) was 0.335. *P*<0.05. n = 42.

S100B protein levels in milk at 3 d after parturition were positively correlated with the maternal post pregnancy BMI (r = 0.335, P = 0.030<0.05) (Figure 2). At one month after parturition, the GDNF levels from mothers who delivered vaginally at term were significantly lower than those who delivered abdominally at term (z = −2.19, P = 0.029<0.05). This correlation persisted until three months after birth (z = −2.17, P = 0.030<0.05). No correlations were found between the levels of other cytokines and age, height, weight, BMI, gestational age, or delivery modes.

**Figure 3:**
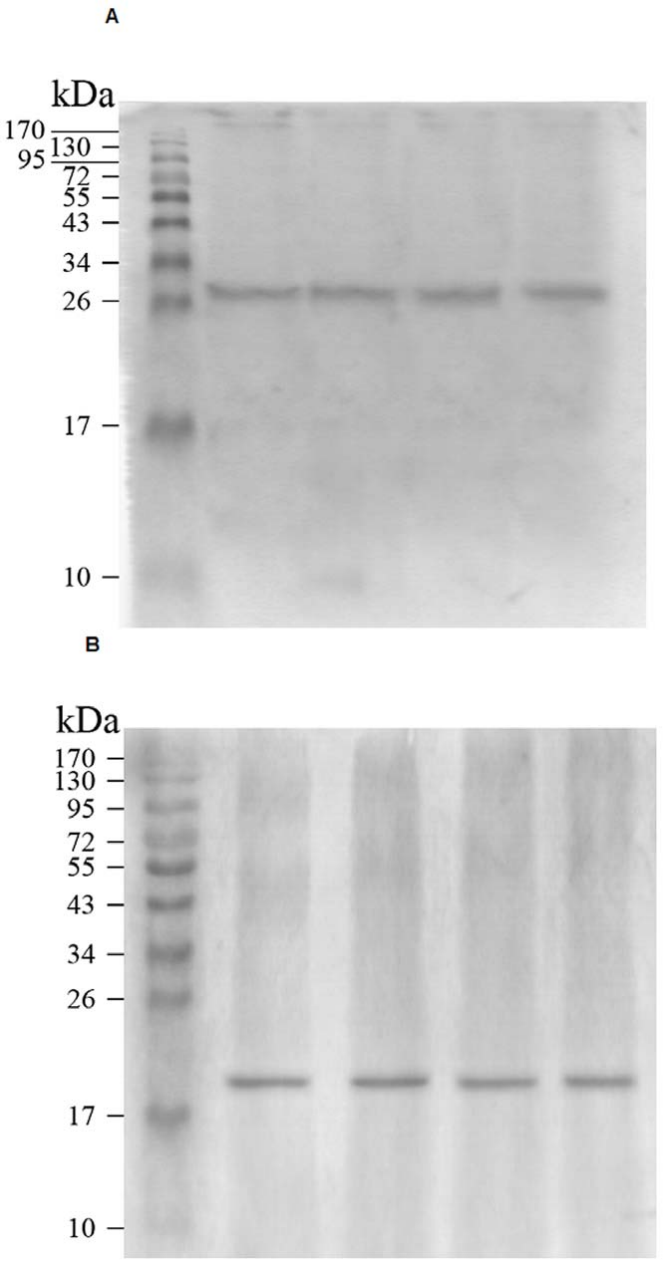
Western blot analysis of BDNF and GDNF in human milk. **A:** BDNF band at approximately 27 kDa. **B:** GDNF band at approximately 20 kDa.

**Figure 4:**
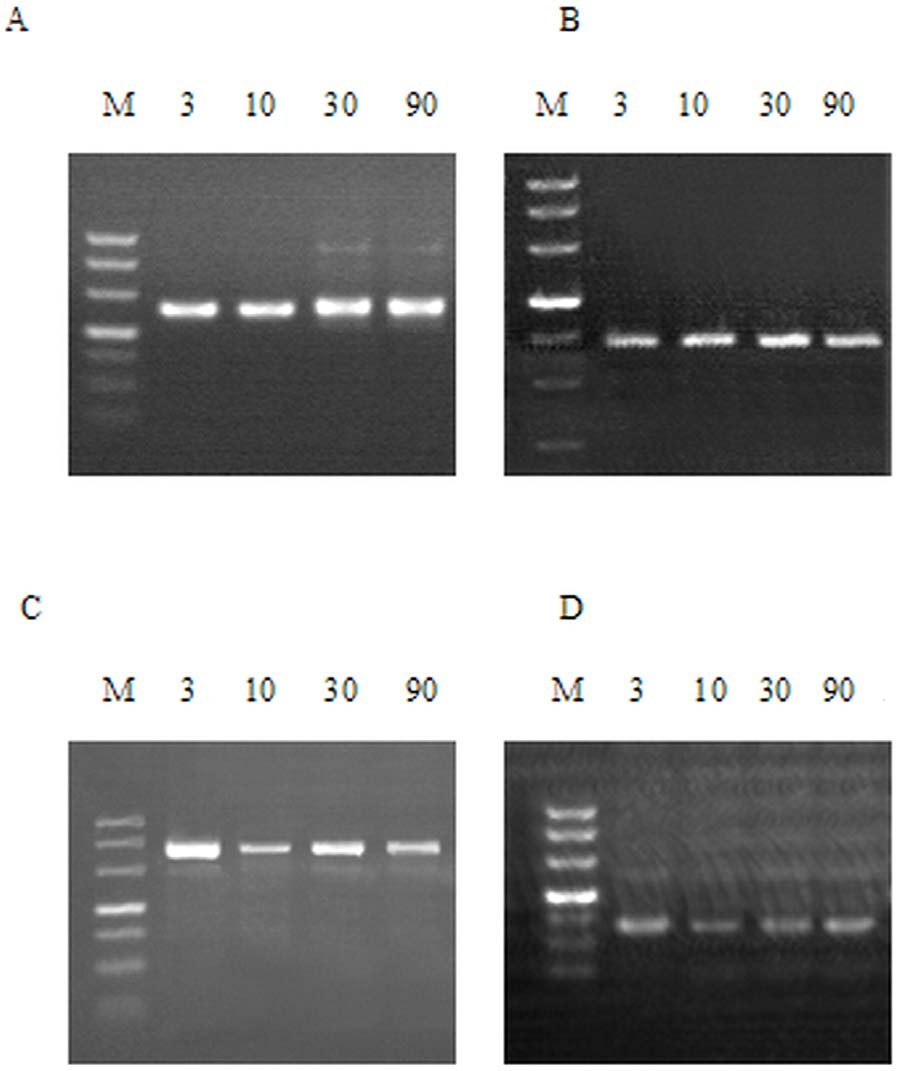
RT-PCR amplified fragments of S100B, BDNF and GDNF mRNA from human milk. M =  DL500 DNA Marker. **A:** bands of β-actin at 256 bp. **B:** bands of S100B at 147 bp. **C:** bands of BDNF at 379 bp. **D:** bands of GDNF at 132 bp. The bands of cytokines from milks collected at day 3, 10, 30, 90 after parturition were not found to vary with time.

Antiserum against BDNF revealed a single band at approximately 27 kDa at all sampling days examined (Figure 3), while the antibody against GDNF labeled a band at approximately 20 kDa (Figure 3). RT-PCR products from milk RNA were subjected to subsequent gel electrophoresis, which showed the expected bands of 147, 379, and 132 bp (Figure 4). Bands of cytokines from milk collected at 3, 10, 30, and 90 d after parturition were not found to vary with time (Figure 4).

## Discussion

This is the first study to report on BDNF and GDNF concentrations in the milk of lactating women. This study investigated changes in these concentrations during lactation, in addition to the measurement of S100B protein in the milk of Chinese women. RT-PCR analysis detected S100B, BDNF, and GDNF mRNA in human milk collected at 3, 10, 30, and 90 d after birth (Figure 4). Western blot analysis was used to confirm the immunoreactivity observed in ELISA assay.

Findings of this study indicate that BDNF and GDNF can be added to the list of bioactive factors (e.g. IL-1b, IL-2, IL-4, IL-5, Lactoferrin, transferrin) [28] present in human milk. S100B protein has been previously documented in human milk at 30 to 929 µg/L, indicating that the lactating human breast secretes S100B protein [29]–[31]. In view of the broad range of S100B protein concentrations reported and the lack of data on human milk from Chinese women, the S100B protein levels in milk from Chinese subjects were quantified in this study. We found that the S100B protein concentrations in milk collected within three months after giving birth is within 390.7–2623.9 ng/L. At day 3 after birth, the S100B protein concentration in milk was much lower than those in milk from Burkinabe and Sicilian women (204.31±63.25 and 199.42±45.28 µg/L, respectively) [31]. However, this does not mean that Chinese infants consume less S100B protein; the overall amount of milk production is independent of ethnicity [31], [32]. S100B, BDNF and GDNF concentrations in milk were much higher than in the serum [33]–[35]. Although the biological significance of the factors in human milk for breastfeeding infants remains unknown, studies suggest that they may serve potential neurotrophic function that may modulate the function and integrity of the GI tract [36], [37] and may exert a stimulating effect on neurodevelopment during breast-feeding or long afterwards [38], [39]. Previous studies have shown that these factors are critical molecules that support the process of neuronal growth, development, protection, and repair [3], [5]–[19], and the modulation of learning and memory [20]–[24]. BDNF plays an important role in the development of the enteric nervous system, defense against intestinal infection, and the modulation of gastrointestinal motility [40], [41]. GDNF has been shown to support the development of human enteric nervous system and intestinal epithelial barrier integrity [42], [43].

Human milk is known to contain leukocytes expressing BDNF and GDNF [44]–[46], which may be reasonably supposed to be the sources of BDNF and GDNF mRNA detected by RT-PCR and of the factors detected by ELISA and Western blot assays. There has been no evidence demonstrating that BDNF and GDNF in human milk are derived entirely from the serum or mammary gland cells. Studies have verified that a significant part of S100B protein present in milk is secreted by mammary epithelial cells and that S100B can be expressed by human milk cells [29].

Detailed information on the fate of these cytokines in the gastrointestinal tract is needed, although we can assume that they participate in the nutritional effects of milk because previous studies have shown that human milk proteins are utilized exceptionally well [47]. Several factors may contribute to these nutritional effects. For instance, human milk contains proteins that bind essential nutrients, thus keeping nutrients in solution and facilitating their uptake by the intestinal mucosa. In addition, protease inhibitors limit the activity of proteolytic enzymes, thereby preserving the physiologic function of some relatively stable binding proteins and some enzymes that can affect the digestion and utilization of macronutrients.

In this study, we found a positive correlation between S100B protein levels in human milk and BMI. This result corroborates previous reports of a direct relationship between S100B serum levels and BMI [48]. More direct evidence of the potential role of S100B in fat metabolism comes from animal studies that have demonstrated the presence of S100B in adipose tissue of rats [49] and that serum S100B levels are significantly influenced by adipose tissue [50].

GDNF levels in milk from mothers who delivered vaginally at term were significantly lower than those who delivered abdominally. This could be attributed to the protective role that breast feeding plays in neonates delivered abdominally. Therefore, cesarean deliveries with no labor complications remain at a much higher risk of neonatal mortality than planned vaginal deliveries [51], because emergency and elective cesarean deliveries are similarly associated with a decreased rate of exclusive breastfeeding compared with vaginal delivery [52].However, no reports have conclusively proven this assumption and it remains to be an important research topic.

This study also found a significant correlation between the levels of S100B protein and BDNF in human milk. At present, no other study had reported this finding, and this association was found in 22 women only. Thus further studies are needed to confirm this relationship.

The present study was constrained by the limited amount of milk samples. Maternal sera were not simultaneously collected due to the peripartum folk customs in China. Furthermore, the absence of previous reports on the basal concentrations of BDNF and GDNF in lactating women prevented comparison of the results. Despite these limitations, the present study was the first to determine basal BDNF and GDNF concentrations in milk from lactating women.

In conclusion, our findings indicate that S100B protein, BDNF, and GDNF are present in human milk. Although their exact functions in human milk are not yet certain, present findings suggest that the physiological function of these cytokines possibly includes a trophic role. The relationship between S100B concentrations in human milk and the BMI of the lactating women has not been described before. This suggests that the S100B protein is a new adipokine. In addition, delivery modes were negatively associated with GDNF concentration in human milk. A positive correlation exists between the levels of S100B protein and BDNF in human milk. Additional investigations are required to clarify the physiologic roles of S100B protein, BDNF, and GDNF in human milk.
